# Trans-arterial radioembolization for intermediate-advanced hepatocellular carcinoma: a budget impact analysis

**DOI:** 10.1186/s12885-018-4636-7

**Published:** 2018-07-05

**Authors:** Carla Rognoni, Oriana Ciani, Silvia Sommariva, Irene Bargellini, Sherrie Bhoori, Roberto Cioni, Antonio Facciorusso, Rita Golfieri, Annagiulia Gramenzi, Vincenzo Mazzaferro, Cristina Mosconi, Francesca Ponziani, Rodolfo Sacco, Franco Trevisani, Rosanna Tarricone

**Affiliations:** 1 Centre for Research on Health and Social Care Management (CERGAS), SDA Bocconi School of Management, Via Roentgen 1, 20136 Milan, Italy; 2 Evidence synthesis and modelling for health improvement (ESMI), University of Exeter Medical School, South Cloisters St Luke’s Campus Exeter, Exeter, UK; 3 College of Public Health, University of South Florida, Tampa, USA; 4 Azienda Ospedaliero-Universitaria Pisana, Via Roma 67, Pisa, Italy; 5 Department of Surgery, Liver Surgery, Transplantation and Gastroenterology, Istituto Nazionale Tumori Fondazione IRCCS, National Cancer Institute, Via G. Venezian 1, Milan, Italy; 6 Azienda Ospedaliero-Universitaria di Bologna, Policlinico S.Orsola-Malpighi, Bologna, Italy; 7 Department of Social and Political Sciences, Bocconi University, Via Roentgen 1, Milan, Italy

**Keywords:** Hepatocellular carcinoma, Trans-arterial radio-embolization, Sorafenib, Budget impact analysis, Costs

## Abstract

**Background:**

Trans-arterial radio-embolization (TARE) is an emerging treatment for the management of hepatocellular carcinoma (HCC). TARE may compete with systemic chemotherapy, sorafenib, in intermediate stage patients with prior chemoembolization failure or advanced patients with tumoral macrovascular invasion with no extra-hepatic spread and good liver function.

We performed a budget impact analysis (BIA) evaluating the expected changes in the expenditure for the Italian Healthcare Service within scenarios of increased utilization of TARE in place of sorafenib over the next five years.

**Methods:**

Starting from patient level data from three oncology centres in Italy, a Markov model was developed to project on a lifetime horizon survivals and costs associated to matched cohorts of intermediate-advanced HCC patients treated with TARE or sorafenib. The initial model has been integrated with epidemiological data to perform a BIA comparing the current scenario with 20 and 80% utilization rates for TARE and sorafenib, respectively, with increasing utilization rates of TARE of 30, 40 and 50% over the next 1, 3 and 5 years.

**Results:**

Compared to the current scenario, progressively increasing utilization rates of TARE over sorafenib in the next 5 years is expected to save globally about 7 million Euros.

**Conclusions:**

Radioembolization can be considered a valuable treatment option for patients with intermediate-advanced HCC. These findings enrich the evidence about the economic sustainability of TARE in comparison to standard systemic chemotherapy within the context of a national healthcare service.

**Electronic supplementary material:**

The online version of this article (10.1186/s12885-018-4636-7) contains supplementary material, which is available to authorized users.

## Background

Liver cancer is one of the most frequent cancers in the world, with a 5-year prevalence of 633,000 cases, 782,000 new diagnoses in 2012, causing more than 700,000 deaths globally per annum [1]. Hepatocellular carcinoma (HCC) is the most frequent type of liver cancer, accounting for 90% of all liver cancers [2].

In order to guide the therapeutic approach and to predict the prognosis of patients with HCC, different staging systems are used. The BCLC (Barcelona-Clinic Liver Cancer) classification is considered the standard system by the American Association of for the Study of Liver Disease (AASLD) [3] and European Association for the Study of the Liver [4]. The system identifies patients with early HCC (stage 0 and A), intermediate (stage B) or advanced stage (stage C) and those with very poor life expectancy (stage D).

Treatment schedules are recommended for each stage, ranging from curative therapies, such as resection or transplant for early stage patients, to best supportive care for terminal patients.

Intra-arterial transcatheter embolotherapies are recommended for non-surgical patients in the intermediate HCC stage, while sorafenib is the standard systemic herapy for patients with advanced HCC and well-preserved liver function and those with intermediate-stage HCC who progress following trans-arterial chemoembolization (TACE). However, in a sub-analysis of trials involving sorafenib, the tolerability of this treatment resulted suboptimal [5]. This situation opened the way to new therapies for the management of intermediate or advanced-stage HCC and, in this setting, transarterial radioembolization (TARE) showed to be a valuable therapeutic option [6–10].

TARE is one type of intra-arterial brachytherapy used to treat HCC. TARE is performed using glass (TheraSphere®, MDS Nordion Inc.) or resin (SIR-Spheres®, Sirtex Medical Inc.) microspheres including β-emitter Y-90. The potential clinical benefits of TARE for the treatment of HCC are under investigation. Its organizational and economic impacts should also be carefully evaluated as TARE is a complex and expensive intervention.

One of the main issues in the evaluation of the cost-effectiveness of locoregional treatments for HCC is the lack of published randomized clinical trials’ results. Decision models may help filling this gap since they allow pooling information from different sources to perform cost-effectiveness analyses. In the literature few studies using this approach are reported. Chaplin and colleagues [11] performed a cost-effectiveness analysis of TARE compared to sorafenib for the treatment of HCC in the UK. The study showed that TARE yielded a total lifetime cost lower than sorafenib (£21,441 vs. £34,050) with a quality adjusted life year (QALY) gain of 0.27 (TARE dominant). Another study assessed the cost-effectiveness of TARE in comparison to conventional transarterial chemoembolization in the United States [12], showing lifetime costs of $31,000 and $48,000 for unilobar and bilobar radioembolization, respectively. For advanced stage patients, the incremental cost-effectiveness ratio of TARE versus TACE resulted 360$ per month (3120$ per year). A more recent study performed in Italy [13] reported for intermediate stage patients an incremental cost-utility ratio (ICUR) for TARE vs. sorafenib of 3302€/QALY, whilst for advanced stage patients, TARE seemed to be a dominant strategy (lower costs and greater health improvements) compared to sorafenib.

But what does this evidence bring to the financial prospects of the national healthcare budget? Although few studies assessed the cost-effectiveness of TARE in comparison to other locoregional or systemic treatments, the budget impact analysis (BIA) of the introduction of this technology at national level is still unexplored. The BIA is an essential component of a complete economic assessment of health technologies aiming at estimating the financial consequences, in the short-medium term, on the total healthcare national budget derived from the diffusion of a new therapeutic intervention, in combination with treatments already used for the management of a particular disease within a specific healthcare system [14].

The cost-effectiveness model developed by Rognoni et al. [13] has been applied to perform a BIA considering increasing utilization rates of TARE in place of sorafenib for the treatment of intermediate-advanced stage HCC patients in the Italian healthcare system over a 5-years horizon.

## Methods

### Markov model

Patient level data were collected at three Italian oncology centers which have performed TARE procedures since 2005: National Cancer Institute, Milan (288 TARE and 125 sorafenib patients); Azienda Ospedaliero-Universitaria Pisana, Pisa (38 TARE and 42 sorafenib patients); Azienda Ospedaliero-Universitaria, Bologna, Policlinico S.Orsola-Malpighi, Bologna (63 TARE and 74 sorafenib patients).

A propensity score matching procedure was performed to obtain two sets of patients, treated with TARE (154 patients) or sorafenib (154 patients), with similar clinical characteristics in terms of Child-Pugh Score, number of nodules (one nodule vs. multinodular) and presence/absence of portal vein thrombosis (PVT). Additional file 1: Table S1 reports the patients’ clinical characteristics before and after the matching.

**Fig. 1:**
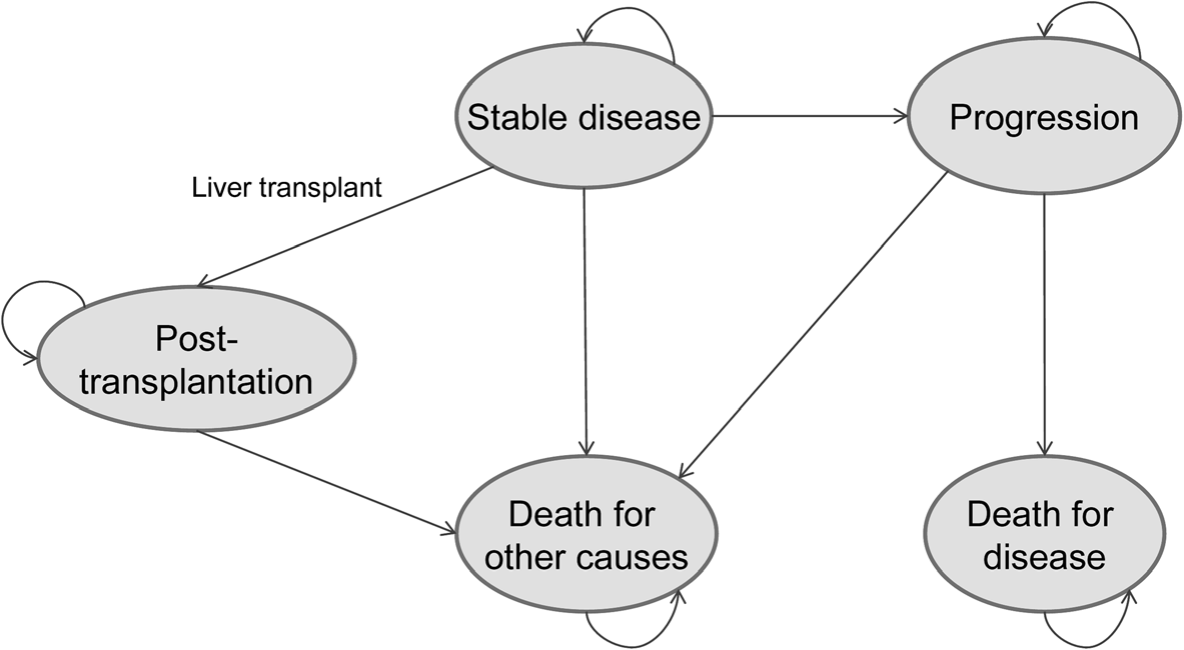
Markov model. A hypothetical cohort of 68-year-old (i.e. mean age of the matched population) HCC patients enter the Markov process in the stable disease state, i.e. with stable HCC

A Markov model was developed to project, on a lifetime horizon, survivals and costs associated to the matched cohorts of intermediate-advanced HCC patients treated with TARE or sorafenib. The health states considered were stable disease, disease progression and death. In the intermediate stage an additional state was included to take into account the possibility of liver transplantation (Fig. 1). A hypothetical cohort of HCC patients enters the Markov process in the stable disease state, i.e. with stable HCC. A focus group with seven expert physicians from the three oncology centers was organized to get their advice on the model structure and validation.

Transition probabilities among different health states were derived from overall survival and progression-free survival curves calculated from the matched cohorts (see Additional file 2: Figure S1). In order to adopt the lifetime horizon in the health economic evaluation, as suggested by NICE [15], original Kaplan-Meier curves were fitted by analytic functions.

In the final matched cohorts, intermediate stage patients undergoing TARE (71%) and sorafenib (49%) reported mean survivals of 24.0 months (median 18.5) and 18.4 months (median 13.0), respectively. In the group of advanced stage patients (29% TARE and 51% sorafenib) these values decreased respectively to 14.9 months (median 11.2) and 16.1 (median 11.3) months.

### Healthcare resources consumption and costs

The analysis was performed from the Italian Healthcare Service perspective and only healthcare costs (€, 2018) incurred by the Healthcare Service were considered. Given the decentralized nature of the Italian healthcare system, with 19 Regions and 2 Autonomous Provinces, for the scope of the present study we referred to costs and tariffs from Lombardy Region, which is the region with the highest DRG reimbursement for TARE. This choice allows for conservative estimates of the hypothetical savings at national level once and if the diffusion of this innovative technology takes place in the clinical practice.

Treatment protocols for TARE and sorafenib were identified through discussions with focus group members. TARE protocol includes a first oncology visit, a procedure simulation (DRG 203), lab exams, and the treatment itself. Admissions for brachytherapy or radioembolization therapies for malignant hepatocellular carcinoma refer to “DRG 409 - Radiotherapy”, with an increased tariff in order to recover the costs related to the microspheres, the interventional radiology procedure and the necessary hospitalization days.

After one month, oncology visit and lab exams are repeated, including an abdomen computed tomography (CT) examination. The follow-up phase includes an oncology visit, lab exams and an abdomen CT examination every three months [16].

The mean number of TARE treatments per patient resulted 1.1 and 1.02 for intermediate and advanced stages, respectively.

Sorafenib is generally delivered monthly (112 cp 200 mg each, hospital cost 3536.17€), after an oncology visit and lab exams, to each patient until disease progression. A payment-by-result scheme is adopted in Italy whereby for each non-responder in the first two months, the drug manufacturer refunds the sustained initial treatment cost (max 2 packages). In order to verify the state of the disease, an abdomen CT examination is performed after two months of treatment with sorafenib, while the rest of the follow-up is the same as for TARE. The mean duration of sorafenib treatment resulted 7.5 and 8.1 months for intermediate and advanced stages, respectively.

Second-line treatments were identified from the matched databases and were included in the model. Liver decompensation was acknowledged by the focus group as the leading side effect, taking into account both economic and clinical aspects. It was assumed that liver decompensation happens in the first year of treatment leading always to hospitalization. As regards liver transplantation, the cost was estimated through the DRG reimbursement tariff, while the yearly cost after the intervention was derived from a study by Cammà and colleagues [17] and inflated to year 2018 using the Italian Consumer Price Index (ITCPI 2005) [18]. All cost components used in the model are summarized in Table 1.

**Table 1:** Healthcare resources and costs used in the model (visits and exams refer to both TARE and sorafenib treatments)

Exam/procedure/DRG	Timing	Code	Cost (€)	Reference
CT examination (abdomen)	every 3 months	88.01.5	137.23	Regional Healthcare Service price list
First visit	1 time	89.7B.6	22.50	Regional Healthcare Service price list
Control visit	every 3 months	89.01.F	17.90	Regional Healthcare Service price list
Blood count	every 3 months	90.62.2	4.05	Regional Healthcare Service price list
Creatinine	every 3 months	90.16.3	1.70	Regional Healthcare Service price list
Sodium	every 3 months	90.40.4	1.70	Regional Healthcare Service price list
Potassium	every 3 months	90.37.4	1.70	Regional Healthcare Service price list
Calcium	every 3 months	90.11.4	1.70	Regional Healthcare Service price list
Prothrombin time	every 3 months	90.75.4	2.60	Regional Healthcare Service price list
Albumin	every 3 months	90.05.1	2.90	Regional Healthcare Service price list
Bilirubin	every 3 months	90.10.4	1.70	Regional Healthcare Service price list
Alpha-Fetoprotein	every 3 months	90.05.5	11.05	Regional Healthcare Service price list
Alanine amino transferase (alt) (gpt)	every 3 months	90.04.5	1.70	Regional Healthcare Service price list
Gamma-glutamyl transpeptidase	every 3 months	90.25.5	1.70	Regional Healthcare Service price list
Alkaline phosphatase	every 3 months	90.23.5	1.70	Regional Healthcare Service price list
Sorafenib	7.5 and 8.1 months duration for intermediate and advanced stages, respectively. Following TARE: 30.3% of patients in intermediate stage, 20% of patients in advanced stage		3787	Monthly Hospital cost
TARE simulation	1 procedure per patient	203	4052	Regional DRG reimbursement
TARE	1.1 procedures per patient in intermediate stage, 1.02 procedures per patient in advanced stage	409	9510	Regional DRG reimbursement
TACE	Following TARE: 18.3% of patients in intermediate stage, 2.2% of patients in advanced stage; Following Sorafenib: 6.6% of patients in intermediate stage, 2.6% of patients in advanced stage	203	4052	Regional DRG reimbursement
RFA/PEI or liver resection	Following TARE: 5.5% in intermediate stage; Following Sorafenib: 10.5% of patients in intermediate stage, 1.3% of patients in advanced stage	192	7549	Regional DRG reimbursement
Radiotherapy	Following TARE: 2.2% in advanced stage	409	4041	Regional DRG reimbursement
Hospitalization for liver decompensation	TARE: 19.4% of patients in intermediate stage, 43% of patients in advanced stage; Sorafenib: in intermediate stage 17.4%, in advanced stage 31%	464	1688	Regional DRG reimbursement
Liver transplantation	Following TARE: 3.7% of patients in intermediate stage	480	68,027	Regional DRG reimbursement
Liver transplantation (yearly cost after intervention)	Following TARE: 3.7% of patients in intermediate stage		6229	Cammà 2013 [17], uplifted to year 2018

### Budget impact analysis

In order to evaluate the expected changes in the expenditure for the Italian Healthcare Service in the hypothesis of an increased utilization of TARE in place of sorafenib, a budget impact model was built based on the initial Markov model. The analysis was conducted in accordance with the ISPOR Principles of Good Practice for Budget Impact Analysis [14]. The Consolidated Health Economic Evaluation Reporting Standards (CHEERS) checklist [19] is reported in the Additional file 3: Appendix.

The model has been developed according to the following steps:

1. Research and analysis of epidemiological data (i.e. incidence) relating to patients with intermediate-advanced stage HCC in Italy, eligible to either TARE or sorafenib,
2. Definition of the current scenario of distribution of patients among the two alternative therapeutic approaches,
3. Definition of future scenarios with appropriate increased use of TARE over sorafenib, considering different annual penetration rates.

The annual incidence of liver carcinoma in Italy accounts for about 13,200 patients [20], of which about 75% can be considered HCCs [21]. Of these, 45.4% (14.9% intermediate + 30.5% advanced) are intermediate or advanced stage tumors according to ITALICA (ITAlian LIver CAncer) database [22].

The counts of intermediate-advanced HCC patients eligible for TARE or sorafenib have been estimated from the ITALICA database taking into account: intermediate stage patients treated with sorafenib (9.5%) and advanced stage patients treated with TACE (28.8%). All together, these account for 10.2% of HCC patients eligible for TARE or sorafenib, that is 1010 intermediate-advanced patients per year in Italy (i.e. 140 intermediate stage and 870 advanced stage).

The current scenario of patients’ distribution between the two alternative treatments was estimated from the ITALICA database as well. Between 2010 and 2014, the registry reported prescription of sorafenib in 9.3% of cases and administration of other treatments (TARE) in 2.1% of cases, therefore the resulting utilization rates of TARE and sorafenib were about 20 and 80%, respectively.

In order to estimate the prevalent patients population (cohort of alive patients) treated with TARE or sorafenib in the current scenario, a simulation was performed using the Markov model, by considering yearly incident cohorts of 1010 patients (140 intermediate stage and 870 advanced stage). A steady prevalent population of 1019 patients resulted considering 20 yearly incident cohorts. In the current scenario the majority of patients (86%) is in the advanced stage and is treated with sorafenib; advanced HCC patients undergoing systemic chemotherapy have a median survival of about 1 year (see Additional file 1: Table S1) and this is the reason why prevalence (1019 patients) and incidence (1010 patients) are similar.

Future scenarios, in which reasonable increased proportions of TARE over sorafenib are considered, were recommended by the focus group as 30, 40 and 50% for 1-year, 3 years and 5-years horizon, respectively. The model applies the variations of the market share to the new incident cohorts (naive treatments), without involving the prevalent cohorts in the variations. Constant incident cohorts were considered in the analysis.

The costs for current and future scenarios were estimated by multiplying yearly costs of each option by the proportion of the eligible population using that option and by the number of patients in the eligible population, taking into account baseline prevalent patients and subsequent yearly incident cohorts. As the focus was on the expected budget at each point in time, the financial streams were presented as undiscounted costs [14].

The flexibility of the model chosen allowed for additional analyses. A number of scenario analyses have been performed to test the robustness of the Markov model results on a lifetime horizon [13]. As the mean number of TARE procedures per patient (from 1 to 3) and sorafenib cost resulted as parameters highly affecting ICER variations [13], budget impact scenario analyses were therefore performed considering 1.5 TARE per patient as reported by [23], and halved sorafenib cost. Moreover, considering increased uses of TARE in the next years, the number of deaths avoided and the number of hospitalizations due to liver decompensation have been estimated.

## Results

### Budget impact analysis

In the intermediate stage, a mean lifetime cost (undiscounted) per patient of 33,040€ (std. dev. 32,766€) and 29,935€ (std. dev. 29,410€) for TARE and sorafenib regimens, respectively, was estimated. These values changed to 22,526€ (std. dev. 11,249€) and 31,526€ (std. dev. 30,930€) for advanced stage patients. These costs include the cost for the treatment itself (TARE or sorafenib), for control visits and examinations, subsequent treatments and for the management of adverse events (liver decompensation). When considering a time horizon of 5 years, in the intermediate stage, costs per patient of 28,003€ (std. dev. 18,217€) and 29,716€ (std. dev. 29,254€) were obtained for TARE and sorafenib regimens, respectively, leading to an incremental cost of 1713€. For advanced stage patients, these values changed to 21,456€ (std. dev. 7399€) and 31,430€ (std. dev. 30,338€), respectively, leading to an incremental cost of 9974€.

Detailed yearly costs by categories are reported in Additional file 4: Table S2.

**Fig. 2:**
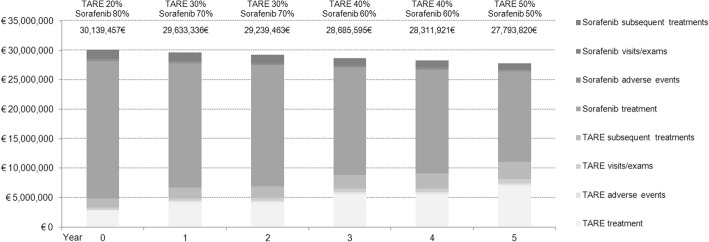
Budget impact for the Italian Healthcare Service considering increasing uses of TARE in the next years

The financial impact on the national healthcare budget after increasing TARE utilization for patients with intermediate-advanced HCC stage was studied. The yearly total cost for TARE and sorafenib treatment mix is presented in Fig. 2. According to the hypothesis that the population is mainly composed by individuals with advanced stage disease and for these patients the cost for TARE is lower in comparison to sorafenib, the first most important observation is that the total budget decreases over time according to the increased use of radioembolization. In comparison to the current scenario (year 0), by progressively increasing TARE utilization rates to 30, 40 and 50%, it would be possible to save 506,121€, 899,993€, 1,453,861€, 1,827,536€ and 2,345,636€ for 1, 2, 3, 4 and 5-year scenarios, respectively, yielding total savings of about 7 million Euros.

**Fig. 3:**
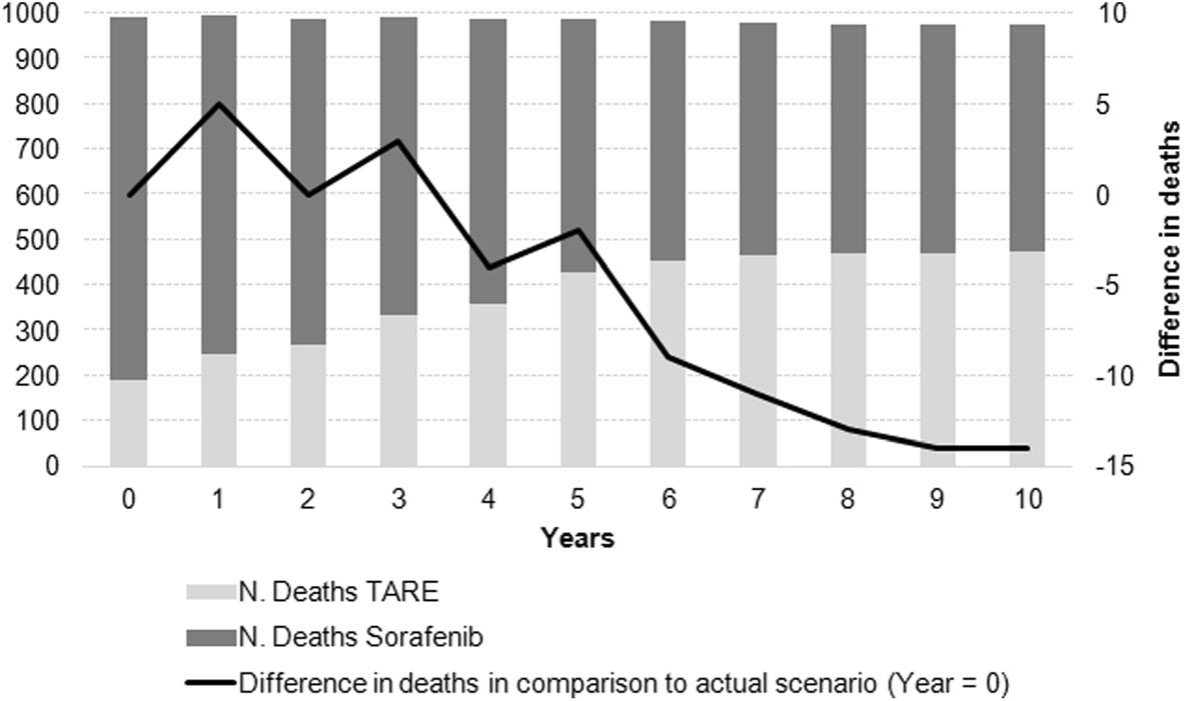
Estimated number of deaths per year for current and future scenarios

**Fig. 4:**
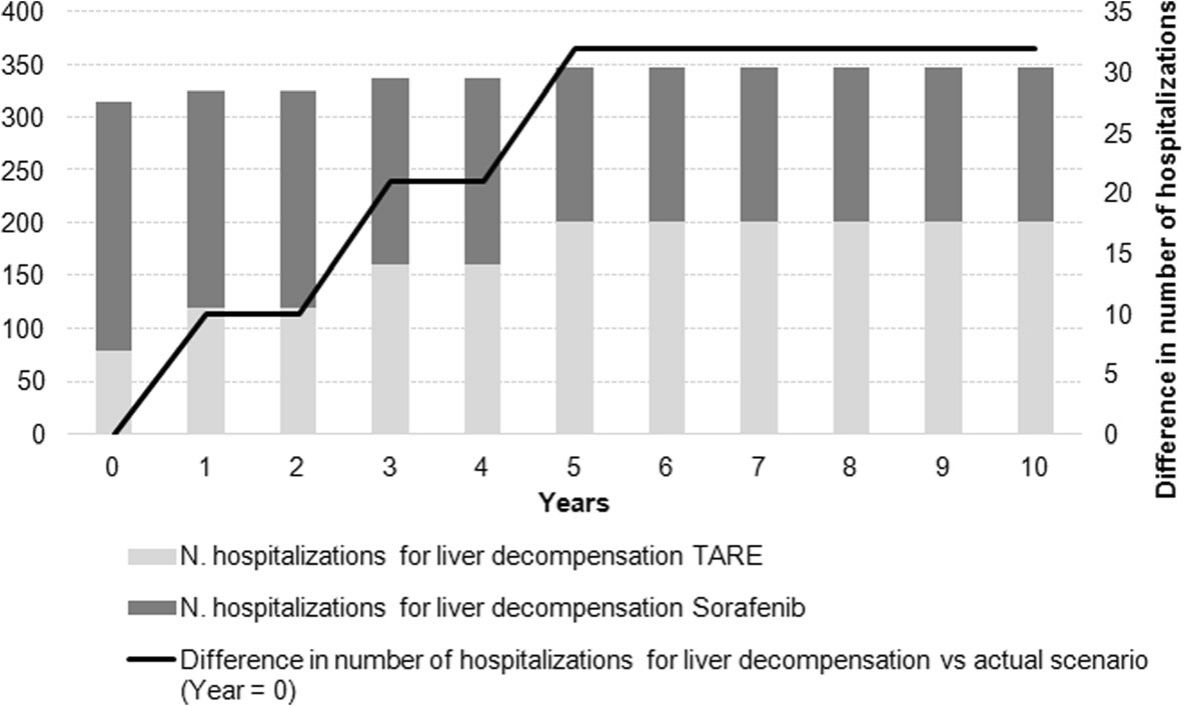
Estimated number of hospitalizations for liver decompensation per year for current and future scenarios

Figure 3 reports, for each year, the number of deaths avoided starting from the current scenario and considering increased uses of TARE in the following years. A time horizon of 10 years has also been investigated for these analyses on expected impact on outcomes to account for the achievement of a “steady state” (with 50% TARE and 50% sorafenib utilization rates from year 5 onwards). As reported in Fig. 3, two is the number of avoided deaths reached at 5 years, but this value increases to fourteen at ten years, when the utilization rate of TARE is maintained at 50%. Using the same approach, Fig. 4 reports the number of additional hospitalizations due to liver decompensation considering increased uses of TARE in the next years. The increase in the number of hospitalizations is due to the higher frequency of liver decompensation for both intermediate and advanced stage patients undergoing TARE in comparison to sorafenib. As described previously, the difference in number of hospitalizations reaches the steady number of 32 from year 5 onwards, when the utilization rates are maintained at 50% for TARE and 50% for sorafenib.

The scenario analysis performed considering a mean number of TARE per patient equal to 1.5 for both intermediate and advanced stages, instead of 1.1 and 1.02, showed total savings of about 1,150,000€ instead of about 7 million Euros. When considering halved sorafenib cost, the analysis led to a total additional cost of about 4,800,000€ over the next five years.

## Discussion

HCC is a life-threatening disease. Despite considerable improvements in the diagnoses and treatments, the available cure options are only partially effective [24] and the disease is very difficult to control when presenting in the advanced stage [3, 25]. Within a resource-limited healthcare system, this context highlights the need for resources to be used efficiently in order to guarantee the best outcomes to this population.

Real world clinical data of two cohorts of patients, treated with TARE or sorafenib, matched according to Child-Pugh score, presence or absence of PVT and number of nodules, were used to populate a model in order to estimate lifetime costs and health outcomes. In the final matched cohorts, intermediate stage patients undergoing TARE and sorafenib yielded mean survivals of 24.0 (median 18.5) and 18.4 months (median 13.0), respectively. In the group of advanced stage patients these values decreased respectively to 14.9 (median 11.2) and 16.1 (median 11.3) months. Patients’ survivals in the TARE group appear lower than other published figures data [9, 26, 27]. The propensity score matching selected pairs of patients with similar clinical characteristics, eligible indifferently to TARE or sorafenib treatments, favoring the selection of patients in worse clinical conditions and, indeed, with lower life expectancy. As regards the costs, the evaluation focused on first-line treatments, control visits and examinations, management of side effects (liver decompensation) and subsequent treatments (second line treatments after TARE or sorafenib).

The budget impact analysis showed that the Italian Healthcare Service could save about 7 million Euros in the hypothesis of an increased utilization of TARE, from 20 to 50%, in place of sorafenib in the next 5 years. The model robustness has already been tested performing a number of sensitivity analyses [13], however, two scenario budget impact analyses showed reduced savings (about 1,150,000€) in case of 1.5 TARE treatments performed per patient or an increase in the national healthcare budget (about 4,800,000€) in case of halved sorafenib cost. It should be noted that these scenarios are unlikely to be representative of the actual practice but can give information on the budget variations in extreme cases.

Budget impact analysis is an important component of the economic evaluation of healthcare interventions, which is gaining significant relevance in formal health technology assessment systems in place across several jurisdictions and certainly in Italy. This is the first study estimating the impact on the Italian healthcare budget of TARE and sorafenib strategies in intermediate-advanced stage HCC patients. A previous study tried to evaluate the patterns of treatment and costs related to HCC treatments, reporting an overall expenditure of 12,215€ for sorafenib and 26,106€ for TARE patients per year. However that analysis was performed from the hospital perspective and based on structured interviews with physicians in four Italian centers [23]. Another study [28], presented the results of a BIA from a hospital perspective in Canada. This study showed that, for a hospital managing 200 HCC patients annually, an increased use of TARE over TACE and sorafenib could incur savings of approximately $37,000, $55,000 and $75,000 in years 1, 2 and 3, respectively.

This study has some limitations. First of all, costs were estimated from the Lombardy Region perspective, and then considered as a proxy for the other Italian Regions. In a decentralized system such as the Italian NHS, one should consider separately each different Region to entirely capture all the specific features of the healthcare service provision and costs [29]. However, most of Italian Regions performing TARE have a special DRG reimbursement rate according to the use of Y-90 microspheres (DRG 409) and in these regions TARE reimbursement tariffs are quite similar (e.g. 8500€ for Emilia Romagna, 8568€ for Piedmont, 9510€ for Lombardy) meaning that the model could be considered reasonably conservative in this respect. Moreover, the healthcare resource consumption has been expressed in natural units (Table 1), as suggested by EunetHTA [30], to allow the model extension to other countries. Furthermore, the budget impact does not take into account the sunk costs of setting up the TARE procedure in a new organization. However, provided the angiographic room and imaging equipment are already available, these costs will refer mainly to a thorough training of the staff [31].

The consumption of healthcare resources has been retrieved from clinical data only for cancer related therapies (i.e. duration of treatment with sorafenib, mean number of TARE treatments per patient and subsequent treatments) while a predefined schedule, although validated by the focus group, for visits and examinations was applied for the follow-up period. This approach might have underestimated the real healthcare resource consumption. Analysis of data on best supportive care offered to patients after treatment failure resulted mainly in the use of off-label drugs or chemotherapy, with no reported indication of dose and duration, and an evaluation including this aspect was not possible. Even though liver decompensation was highlighted by the focus group as the main and most expensive adverse effect caused by TARE or sorafenib, other adverse events could have an impact on both costs and patient’s quality of life. More data on the occurrence of other side effects would allow for a more comprehensive evaluation.

As regards epidemiological data on prevalence and incidence of HCC in intermediate and advanced eligible for TARE and sorafenib therapies, as well as the current mix of treatment strategies in the eligible population, we relied on the largest source in Italy, the ITALICA registry, reporting data updated to 2014. Considering that TARE is an emerging treatment showing a promising efficacy in terms of disease control with a good safety profile [32], it is likely that its diffusion has been underestimated. In this regard, the results shown may have overestimated the potential savings due to the diffusion of this advanced medical device technology occurred since the last update of the ITALICA registry (2014).

## Conclusions

Radioembolization can be considered a valid treatment option, giving an “additional” chance of survival for patients with intermediate-advanced hepatocellular carcinoma. The attitude towards this type of treatment is usually positive, while eventual side effects are considered tolerable. The present study adds evidence about the economic sustainability of TARE in comparison to standard systemic chemotherapy, sorafenib, at national level, showing that a decrease of the Italian healthcare budget is possible through an increase of the diffusion of this advanced medical device technology. Further prospective studies and increased awareness around the cost-effectiveness profile of healthcare technologies in this area will be able to provide additional data to confirm our conclusions.

## Additional files

Additional file 1: **Table S1.** Characteristics of unmatched and matched treatment groups; The table shows the characteristics of patients who underwent TARE or sorafenib in unmatched and matched cohorts. (DOCX 14 kb)

Additional file 2: **Figure S1.** Model curves fitting for PFS and OS; The figure reports model curves fitting (continuous lines) for PFS (A: intermediate stage, B: advanced stage) and OS (C: intermediate stage, D: advanced stage). TARE strategy is represented in blue while sorafenib in red. Segmented lines represent the original data. (TIF 396 kb)

Additional file 3: Appendix. CHEERS Checklist; The ISPOR CHEERS (Consolidated Health Economic Evaluation Reporting Standards) checklist shows recommended items to be included in reports of economic evaluations of health interventions. (PDF 108 kb)

Additional file 4: **Table S2.** Mean annual costs per patient for intermediate and advanced stages HCC related to TARE and sorafenib treatments; The table reports, for TARE and sorafenib, mean annual costs per patient for the following cost categories: first-line treatment, management of adverse events, visits/exams, subsequent treatments. (DOCX 18 kb)

## Abbreviations

BCLC: Barcelona-Clinic Liver Cancer
BIA: Budget Impact Analysis
HCC: Hepatocellular carcinoma
NHS: National Healthcare Service
PVT: Portal Vein Thrombosis
SIRT: Selective Internal Radiation Therapy
TACE: Trans-Arterial Chemo-Embolization
TARE: Trans-Arterial Radio-Embolization
