# Supplementary material for: Trans-arterial radioembolization for intermediate-advanced hepatocellular carcinoma: a budget impact analysis
Source: BMC Cancer. 2018 Jul 5;18:715. doi: 10.1186/s12885-018-4636-7 (PMC6034232; doi:10.1186/s12885-018-4636-7)
Supplement: Supplementary file 1 — Table S1. Characteristics of unmatched and matched treatment groups; The table shows the characteristics of patients who underwent TARE or sorafenib in unmatched and matched cohorts. (DOCX 14 kb) [file 12885_2018_4636_MOESM1_ESM.docx]

Supplementary Table 1 - Characteristics of unmatched and matched treatment groups

|  | Unmatched cohorts | | | Matched cohorts | | | Test |
| --- | --- | --- | --- | --- | --- | --- | --- |
| Characteristics | TARE | Sorafenib | p | TARE | Sorafenib | p |  |
| Mean age (years) | 66 | 70 | <0.001 | 67 | 70 | 0.02 | T-test |
| Males | 82.1% | 78.5% | 0.3 | 84.4% | 76% | 0.6 | Chi-square |
| Multinodular* | 70.1% | 86.6% | <0.001 | 86.4% | 86.4% | 1 | Chi-square |
| PVT* | 61.7% | 25.6% | <0.001 | 24% | 24% | 1 | Chi-square |
| Child Pugh A* | 92.4% | 92.3% | 0.97 | 95.5% | 95.5% | 1 | Chi-square |

*variable selected for propensity score matching
