# Supplementary figures and images for: Trans-arterial radioembolization for intermediate-advanced hepatocellular carcinoma: a budget impact analysis
Source: BMC Cancer. 2018 Jul 5;18:715. doi: 10.1186/s12885-018-4636-7 (PMC6034232; doi:10.1186/s12885-018-4636-7)

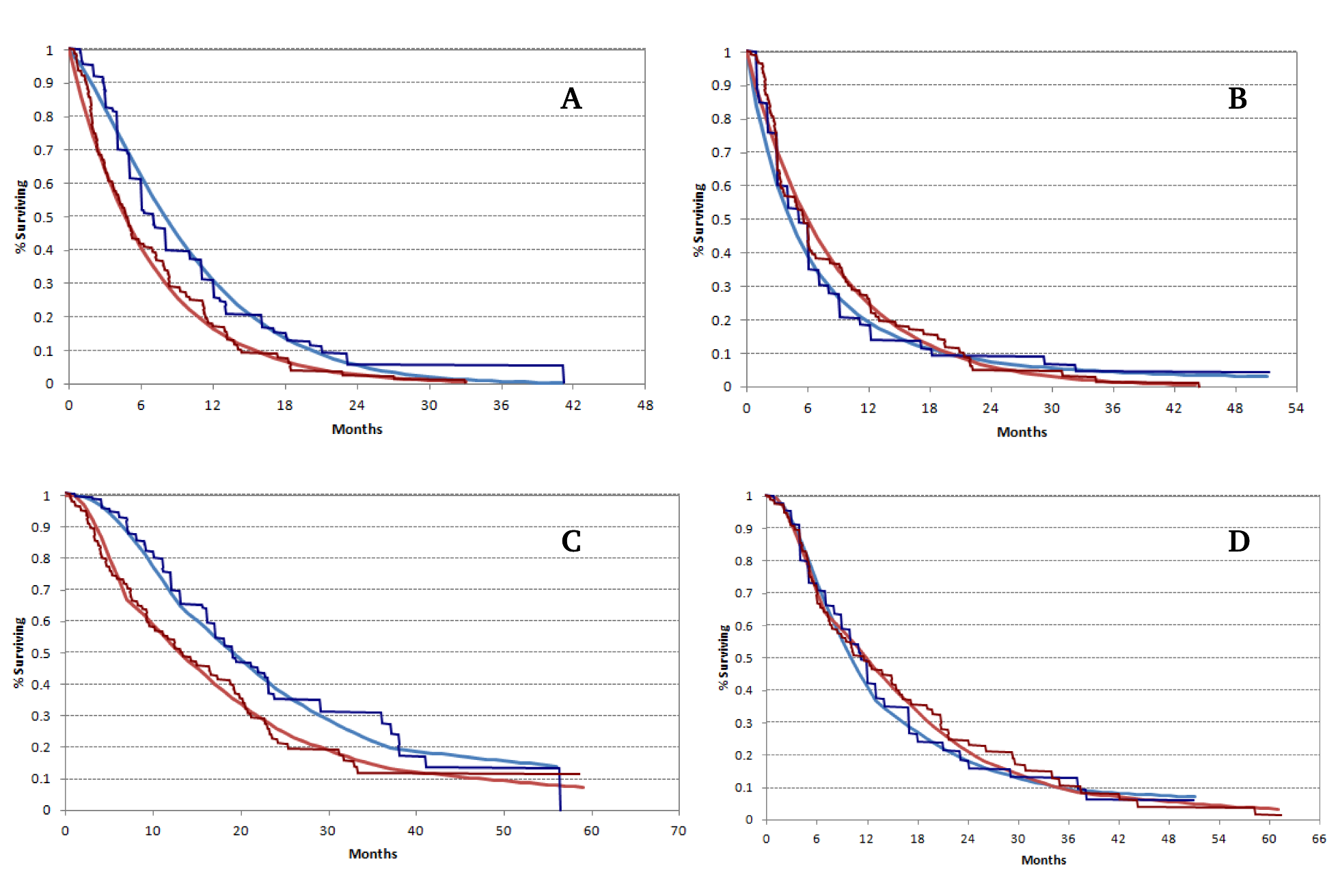

Supplement: Supplementary file 2 — Figure S1. Model curves fitting for PFS and OS; The figure reports model curves fitting (continuous lines) for PFS (A: intermediate stage, B: advanced stage) and OS (C: intermediate stage, D: advanced stage). TARE strategy is represented in blue while sorafenib in red. Segmented lines represent the original data. (TIF 396 kb) [file 12885_2018_4636_MOESM2_ESM.tif]
