# Supplementary material for: Trans-arterial radioembolization for intermediate-advanced hepatocellular carcinoma: a budget impact analysis
Source: BMC Cancer. 2018 Jul 5;18:715. doi: 10.1186/s12885-018-4636-7 (PMC6034232; doi:10.1186/s12885-018-4636-7)
Supplement: Supplementary file 4 — Table S2. Mean annual costs per patient for intermediate and advanced stages HCC related to TARE and sorafenib treatments; The table reports, for TARE and sorafenib, mean annual costs per patient for the following cost categories: first-line treatment, management of adverse events, visits/exams, subsequent treatments. (DOCX 18 kb) [file 12885_2018_4636_MOESM4_ESM.docx]

Supplementary Table 2 – Mean annual costs per patient for intermediate and advanced stages HCC related to TARE and sorafenib treatments

| **Intermediate stage** | | | | | | | | | | |
| --- | --- | --- | --- | --- | --- | --- | --- | --- | --- | --- |
| **Year** | **First-line treatment** | | **Management of adverse events** | | **Visits/exams** | | **Subsequent treatments** | | **Total cost** | |
|  | TARE | Sorafenib | TARE | Sorafenib | TARE | Sorafenib | TARE | Sorafenib | TARE | Sorafenib |
| 1 | € 14,918 | € 21,994 | € 327 | € 294 | € 975 | € 836 | € 4,117 | € 859 | € 20,337 | € 23,983 |
| 2 | € 0 | € 3,879 | € 0 | € 0 | € 533 | € 454 | € 2,815 | € 168 | € 3,348 | € 4,501 |
| 3 | € 0 | € 632 | € 0 | € 0 | € 308 | € 223 | € 1,667 | € 27 | € 1,974 | € 883 |
| 4 | € 0 | € 103 | € 0 | € 0 | € 190 | € 131 | € 1,098 | € 4 | € 1,289 | € 238 |
| 5 | € 0 | € 17 | € 0 | € 0 | € 150 | € 93 | € 904 | € 1 | € 1,054 | € 110 |
| 6 | € 0 | € 3 | € 0 | € 0 | € 118 | € 65 | € 748 | € 0 | € 866 | € 68 |
| 7 | € 0 | € 0 | € 0 | € 0 | € 92 | € 46 | € 622 | € 0 | € 714 | € 47 |
| 8 | € 0 | € 0 | € 0 | € 0 | € 72 | € 32 | € 521 | € 0 | € 593 | € 32 |
| 9 | € 0 | € 0 | € 0 | € 0 | € 56 | € 23 | € 439 | € 0 | € 495 | € 23 |
| 10 | € 0 | € 0 | € 0 | € 0 | € 43 | € 16 | € 373 | € 0 | € 417 | € 16 |
| 15 | € 0 | € 0 | € 0 | € 0 | € 33 | € 11 | € 320 | € 0 | € 353 | € 11 |
| 20 | € 0 | € 0 | € 0 | € 0 | € 25 | € 8 | € 275 | € 0 | € 301 | € 8 |
| **Advanced stage** | | | | | | | | | | |
| **Year** | **First-line treatment** | | **Management of adverse events** | | **Visits/exams** | | **Subsequent treatments** | | **Total cost** | |
|  | TARE | Sorafenib | TARE | Sorafenib | TARE | Sorafenib | TARE | Sorafenib | TARE | Sorafenib |
| 1 | € 13,833 | € 21,732 | € 726 | € 523 | € 867 | € 812 | € 2,557 | € 147 | € 17,983 | € 23,214 |
| 2 | € 0 | € 5,661 | € 0 | € 0 | € 277 | € 404 | € 1,489 | € 42 | € 1,766 | € 6,107 |
| 3 | € 0 | € 1,381 | € 0 | € 0 | € 130 | € 167 | € 704 | € 10 | € 834 | € 1,558 |
| 4 | € 0 | € 336 | € 0 | € 0 | € 77 | € 81 | € 405 | € 2 | € 483 | € 420 |
| 5 | € 0 | € 82 | € 0 | € 0 | € 62 | € 49 | € 329 | € 1 | € 391 | € 131 |
| 6 | € 0 | € 20 | € 0 | € 0 | € 48 | € 29 | € 248 | € 0 | € 296 | € 49 |
| 7 | € 0 | € 5 | € 0 | € 0 | € 38 | € 17 | € 175 | € 0 | € 213 | € 22 |
| 8 | € 0 | € 1 | € 0 | € 0 | € 30 | € 10 | € 122 | € 0 | € 153 | € 11 |
| 9 | € 0 | € 0 | € 0 | € 0 | € 25 | € 6 | € 85 | € 0 | € 110 | € 6 |
| 10 | € 0 | € 0 | € 0 | € 0 | € 21 | € 3 | € 58 | € 0 | € 78 | € 3 |
| 15 | € 0 | € 0 | € 0 | € 0 | € 18 | € 2 | € 39 | € 0 | € 57 | € 2 |
| 20 | € 0 | € 0 | € 0 | € 0 | € 15 | € 1 | € 26 | € 0 | € 41 | € 1 |
